# Experimental and Numerical Study of the Thermo-Viscoplastic Behavior of NICRO 12.1 for Perforation Tests

**DOI:** 10.3390/ma13194311

**Published:** 2020-09-27

**Authors:** Eva Alonso-Elías, Alexis Rusinek, Ignacio Rubio-Díaz, Richard Bernier, Marcos Rodríguez-Millán, María Henar Miguelez

**Affiliations:** 1 SERTEC, SL. (Servicios de Tecnología, Ingeniería e Informática, S.L.) Avda. Rita Levi Montalcini 14, 28906 Getafe, Madrid, Spain; 2 Laboratory of Microstructure Studies and Mechanics of Materials (LEM3), Lorraine University, 7 rue Félix Savart, BP 15082, 57073 Metz CEDEX 03, France; alexis.rusinek@univ-lorraine.fr (A.R.); richardenim@gmail.com (R.B.); 3 Department of Mechanical Engineering, University Carlos III of Madrid, Avda. de la Universidad 30, 28911 Leganés, Madrid, Spain; igrubiod@pa.uc3m.es (I.R.-D.); mrmillan@ing.uc3m.es (M.R.-M.); mhmiguel@ing.uc3m.es (M.H.M.)

**Keywords:** dynamic characterization, failure mode, Johnson–Cook model

## Abstract

Dynamic impact tests using thin metal plates for ballistic characterization have received significant attention in recent years. The Johnson–Cook (J–C) model is extensively used in numerical modeling of impact and penetration in metals. The AISI (American Iron and Steel Institute) 301 steel family presents good impact behavior, excellent formability, and high corrosion resistance. Thus, NICRO (Nickel and Hard Chrome Plated Steel) 12.1 (part of the AISI 301 steel family) was chosen in this work, although parameters of the J–C model or impact results were not found in the literature. In this work, NICRO 12.1 steel plates, were characterized in ballistics with an initial impact velocity up to 200 m/s and three shape nose projectiles. The Johnson–Cook parameters for the NICRO 12.1 steel were calculated for a large range of temperatures and strain rates. Impact tests were carried out using three projectiles: conical, hemispherical, and blunt. The ballistic curves, failure mode, and maximum deformation obtained with each projectile, experimentally and numerically, were compared, and a good correlation was obtained.

## 1. Introduction

The impact performance of protective structures has been a recurrent study subject because of its application in numerous areas. Dynamic impact tests using thin metal plates at room temperature of undeformable projectiles are commonly developed to characterize ballistic performance. Nevertheless, the temperature and the strain rate dependence must be taken into account in armor design [1,2].

Steel plates are used in armor plates because of their favorable properties (high strength, hardness, and moderate ductility) against the impact of projectiles. Plasticity and fracture properties of the material are crucial in the development of numerical models to reduce costs and time from experimental tests [3,4].

The impact behavior of metal plates is a complex problem because it depends on a significant number of parameters of the projectile (mainly projectile nose shape, length, initial impact velocity, diameter, and nose impact angle), the plate (mainly thickness, material hardness, monolithic plate or sandwich configuration), and testing parameters (initial temperature and boundary conditions) [5,6,7]. Several authors, such as P.K. Gupta, T. Borvik, and R.L. Woodward, have carried out significant research in this field [7,8,9].

In contrast, based on the studies of Wierzbicki et. al. [9] and Kepenyigba et al. [5], triaxiality depends on the projectile nose shape. Blunt projectiles produces a tensional state of shearing (triaxiality = 0), conical projectile produces a tensional state by piercing with a triaxiality of 1/3, and hemispherical projectiles produces a mixed tensional state (triaxiality = 2/3). Thus, it has been observed that the failure mode of a thin steel plate presents a strong dependence with the projectile nose shape (triaxiality), in addition to the initial impact velocity ($v_{0}$). Plugging is usually observed in impact tests with blunt projectiles due to shear failure mode, and plastic strain appears in the region close to the impact zone. Petaling is the failure mode usually observed with conical projectiles. Radial necking appears due to the piercing process. Plug ejection and radial cracks with necking appear with hemispherical projectiles due to radial hole expansion, leading to petaling [7,10,11,12].

In the literature, numerous models to predict the plastic response of metals working under dynamic loads can be found. Specifically, the Johnson–Cook (J–C) model is extensively used to develop numerical impact and penetration models in metals. The calibration of the J–C model to obtain parameters is usually based on tests under different strain rates and temperatures. In addition, the J–C model provides good results for a large range of applied loads and elevated temperatures [3,4,13].

Xiao et al. [13] developed a slightly modified Johnson–Cook model for the impact simulations of aluminum 2024-T351 plates with a blunt projectile. Rodríguez-Millán et al. [2] studied the stress state of three aluminum alloys tested under impact loads, and the Johnson–Cook model was used in the simulation of finite elements of the experimental tests. Holmen et al. [10] analyzed the influence of the yield-surface shape in a ballistic impact simulation using a lightly modified J–C model as part of the study. Senthil et al. [14] developed a numerical simulation with a commercial finite element software, ABAQUS/Explicit, using the constitutive Johnson–Cook model to predict the ballistic resistance of mild steel. Rusinek et al. [15] used the Johnson–Cook model to study the penetration of Weldox 460 E plates and the failure mode associated with each projectile nose shape.

Different techniques to obtain model parameters can be found in the literature [4,13]. Farahani et al. [16] carried out experimental tests to obtain the stress–strain curves required to identify the J–C constitutive model parameters, with the exception of one parameter, because a great number of tests are needed. This parameter was obtained by adjusting the numerical simulations to the experimental impact tests carried out with a steel ball. Sobolev et al. [17] developed a procedure to obtain the J–C parameters from the study of three materials at different temperatures and strain rates. In this case, a numerical model was developed with these materials to simulate shipping cask drop tests.

The novelty of the current work is the characterization of the ballistic performance of NICRO 12.1 steel plates at a large range of temperatures and strain rates and the development of a numerical model for further research. The parameters of the J–C model were not found for NICRO 12.1 steel in the literature, and the derivation of these parameters is also a contribution of the present work. Quasistatic tensile tests at room temperature and dynamic compression tests at a wide range of temperatures and strain rates were carried out. These parameters were used on the numerical simulations developed using ABAQUS/Explicit for three different projectiles (blunt, conical, and hemispherical). The numerical results were compared with the experimental results under the same conditions.

## 2. Materials and Methods

### 2.1. Materials

The steel tested in this work is called NICRO 12.1 (supplied by Berndorf Band, Berndorf, Austria), which is part of the AISI 301 and DIN 1.4310 steels families. Its chemical composition is given in Table 1.

AISI 300 family steels exhibit an austenitic structure. When cold deformation and/or heat treatments are applied, the austenitic structure changes into martensite. The percentage of martensite depends in each case on the treatments applied [18,19]. Due to the martensitic transformation process, some authors [1,18] report an improvement of the mechanical properties (i.e., higher elastic limit, increased work hardening rate, and enhanced ductility), whereas others note that other properties (i.e., Young’s modulus) can deteriorate [20].

AISI 301 steels have been widely studied as structural materials for applications, such as military vehicles, due to their capability to manage impact energy and their excellent formability and high corrosion resistance [21]. In addition, mechanical treatments improve the mechanical properties of AISI 301 steel. Its chemical composition is given in Table 2.

The steel used in this work has received cold work and thermal treatment during its processing. Thus, the transformation from austenite to martensite was initiated, and this steel presents 33% of the martensitic structure. Its properties are shown in Table 3.

**Figure 1:**
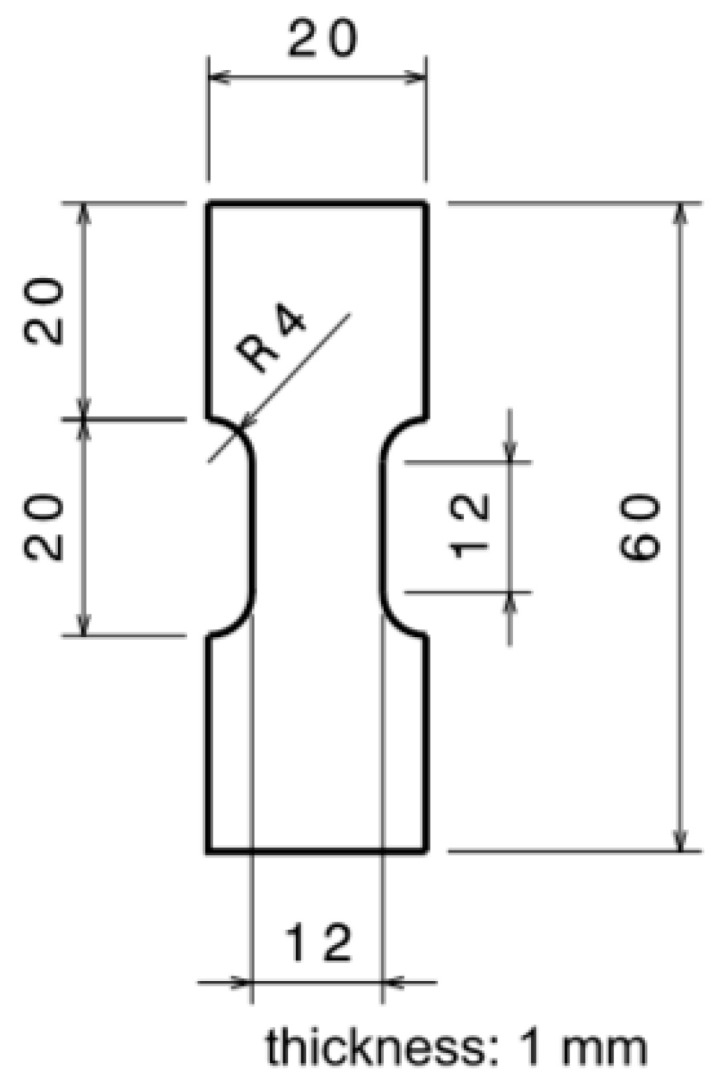
Traction sample geometry (mm).

The starting material consists of NICRO 12.1 steel plates with a thickness of 1 mm. These steel plates were machined by laser to obtain the specimens necessary for traction tests. The geometry is shown in Figure 1. In addition, the specimens for compression were cylindrical samples with diameter of 6 mm and thickness of 3 mm to be used with Split Hopkinson Pressure Bars (SHPBs).

### 2.2. Methods

The tests performed with NICRO 12.1 and the modeling of its thermo-viscoplastic behavior are described below.

#### 2.2.1. Quasi-Static Tensile Tests

The NICRO 12.1 steel was tested under a quasi-static strain rate at room temperature. The geometry of the samples used is shown in Figure 1. The test was repeated four times. The machine used to perform these tests was a Zwick/Roell model (type 1484, ser. No. 94861/04, Fnom 200 kN) provided by ZwickRoell (Ulm, Germany).

**Figure 2:**
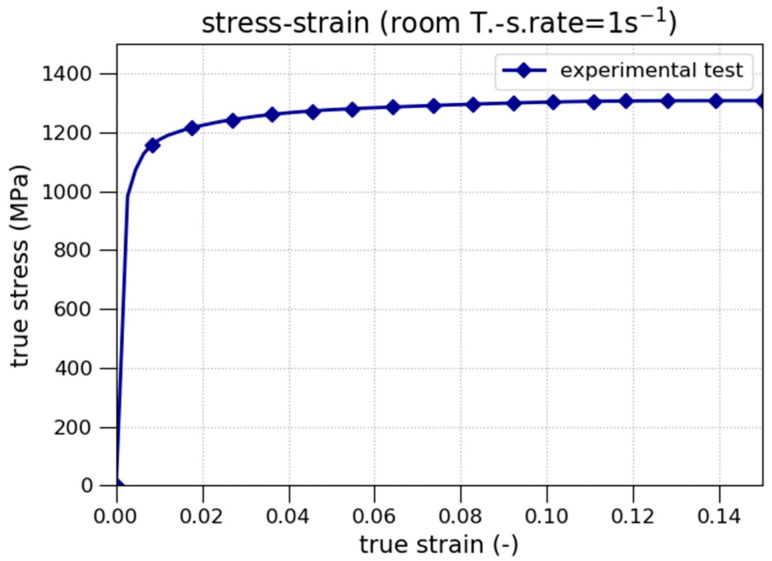
True stress–strain curves at room temperature and the reference strain rate.

The true stress–strain curves obtained from the tensile quasi-static tests performed with NICRO 12.1 steel at room temperature and the reference true strain rate ($\dot{\varepsilon }=1s^{-1}$) are shown in Figure 2. The failure strain is 0.15, and the yield stress obtained is 1055 MPa.

#### 2.2.2. Dynamic Compression Tests

The samples of NICRO 12.1 were also tested under uniaxial dynamic compression at different temperatures (room temperature, and 100 and 200 °C) and three strain rates (2700, 3700, and 4300 $s^{-1}$). For each experimental condition, the test was performed two times. The gas gun (provided by the LEM3, Metz, France) is a conventional Split Hopkinson Pressure Bars (SHPBs), and was used in dynamic compression tests.

**Figure 3:**
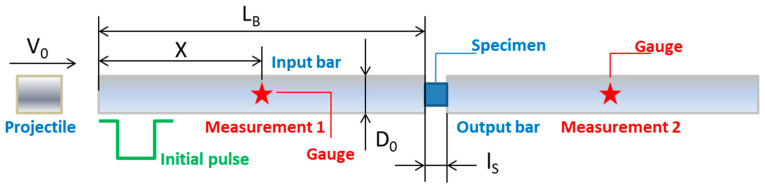
Compression machine set-up.

The dynamic compression tests were carried out using the SHPB. This technique allows the forces applied to the specimens to be obtained using the elastic theory of elastic wave propagation in the bars. The set-up is shown in Figure 3.

The strain rate depends on $v_{0}$ and the yield stress of the material. An incident stress elastic wave, $\sigma_{1}$, is produced when the projectile hits the input bar. $\sigma_{1}$ travels along the impact bar with an elastic wave speed $C_{0}$. It depends on Young’s modulus and the material density *ρ* ($C_{0}=\;\sqrt{E/\rho }$). The incident wave intensity, $\sigma_{1}$, is proportional to $v_{0}$ as follows: $\sigma_{1}=\rho_{0}C_{0}v_{0}/2$. When the incident strain wave $\varepsilon_{I}=\sigma_{I}/E$ reaches the specimen interface, it is partially reflected ($\varepsilon_{R},\;\sigma_{R}$) and partially transmitted ($\varepsilon_{T},\;\sigma_{T}$) along the output bar [22].

Using Equation (1), in which the uniaxial wave propagation and uniform stress distribution in the specimen are assumed, in the case of dynamic forces equilibrium, the stresses, strains, and strain rates can be obtained [22].

(1) $$\begin{array}{c} \sigma \left(t\right)=\;\frac{E_{b}}{2}\frac{\varphi_{b}}{\varphi_{s}}\left(\varepsilon_{I}+\varepsilon_{R}+\varepsilon_{T}\right)\\ \varepsilon \left(t\right)=\;\frac{C_{o}}{L_{o}}\int \left(\varepsilon_{I}+\varepsilon_{R}+\varepsilon_{T}\right)d\psi \\ \dot{\varepsilon }\left(t\right)=\;\frac{C_{o}}{L_{o}}\left(\varepsilon_{I}+\varepsilon_{R}+\varepsilon_{T}\right) \end{array}\}\to \varepsilon_{T}=\varepsilon_{I}+\varepsilon_{R}\to \{\begin{array}{c} \sigma \left(t\right)=\;E_{b}{\left(\frac{\varphi_{b}}{\varphi_{s}}\right)}^{2}\left|\varepsilon_{T}\right|\\ \varepsilon \left(t\right)=\;\frac{2C_{o}}{L_{o}}\int_{0}^{t}\left|\varepsilon_{R}\left(\psi \right)\right|d\psi \\ \dot{\varepsilon }\left(t\right)=\;\frac{2C_{o}}{L_{o}}\left|\varepsilon_{R}\left(t\right)\right| \end{array}$$

The thermo-viscoplastic behavior σ(ɛ) for a specific strain rate ($\dot{\varepsilon }$) can be defined using Equation (1). The WASP (Waves Analysis and Study Program) software is a homemade program developed at the laboratory of LEM3. It allows the average stress-strain curve of the material tested to be derived using the recorded data of the incident, reflected, and transmitted waves. Furthermore, WASP allows corrections on interface friction and adiabatic effects to determine the behavior of the material tested. More information about SHPB testing and the analytical approach presented previously can be found in [1,20].

**Figure 4:**
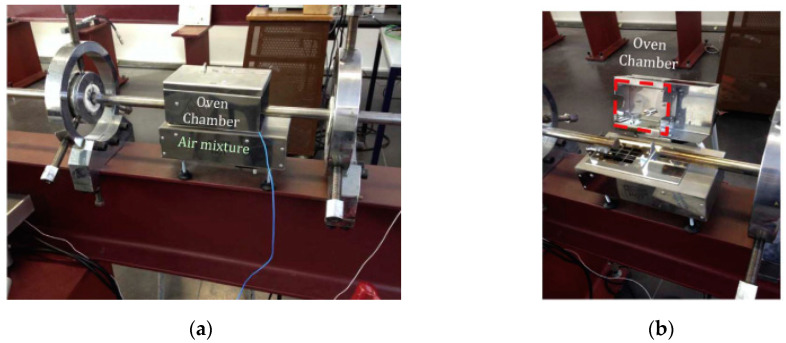
Furnace used in compression machine: (**a**) closed furnace; (**b**) open furnace [23].

To perform tests at higher temperatures, a furnace was coupled to the SHPB (see Figure 4) to keep the testing samples at a constant temperature. It was necessary to wait 30 min to reach a uniform temperature in the sample during the test performance [20,21].

**Figure 5:**
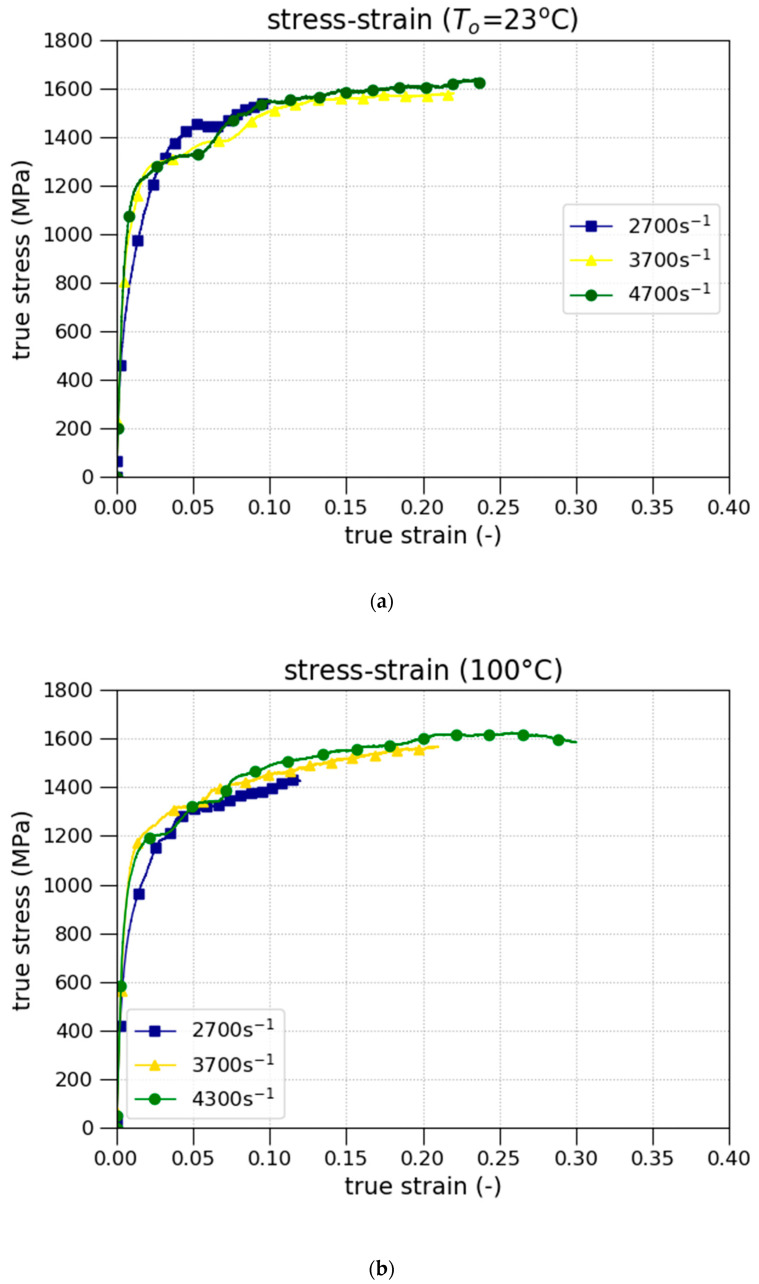

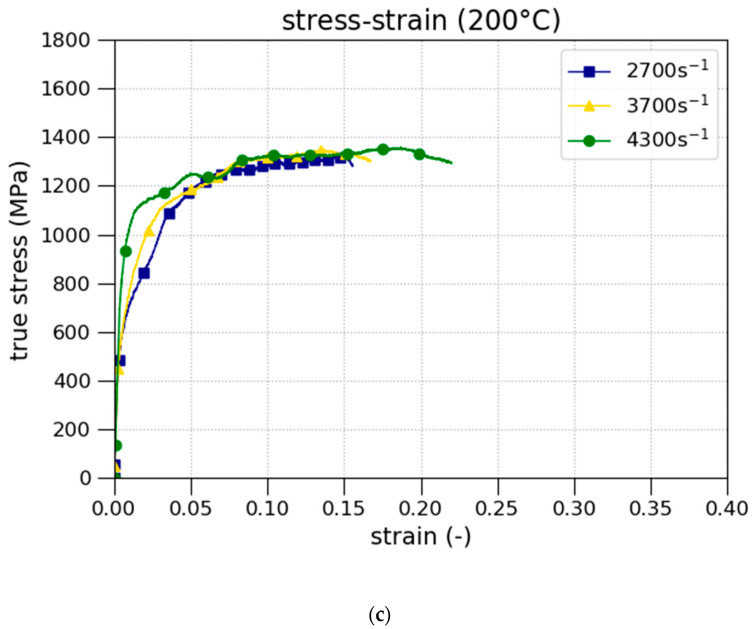
True stress–strain curves at: (**a**) room temperature; (**b**) 100 °C; (**c**) 200 °C.

Figure 5a, presents the true stress–strain curves at room temperature for high strain rates ($2700\;s^{-1}<\dot{\varepsilon }<4300\;s^{-1}$). Figure 5b,c show the true stress–strain curves at 100 and 200 °C, respectively, and high strain rates ($2700\;s^{-1}<\dot{\varepsilon }<4300\;s^{-1}$).

Concerning the yield strength, no temperature sensitivity of the yield stress was observed from the experimental tests performed. However, the change of temperature in Figure 5 shows that the plastic deformation at the fracture point decreases with the temperature and with the strain rate.

#### 2.2.3. Modeling of the Thermo-Viscoplastic Behavior

The Johnson–Cook model is commonly used to express the relationship between stress, strain, and temperature of a metallic material under conditions of large deformation, high strain rate, and elevated temperatures [4]. The flow stress model is expressed, as shown in Equation (2) [24].

(2) $$\sigma \left(\varepsilon^{P},{\dot{\varepsilon }}^{P},T\right)=\left(A+B{\left(\varepsilon^{P}\right)}^{n}\right)\cdot \left(1+C\cdot \log\left(\frac{{\dot{\bar{\varepsilon }}}^{P}}{{\dot{\bar{\varepsilon }}}_{o}}\right)\right)\cdot \left(1-{\hat{T}}^{m}\right)$$

where:

$\varepsilon^{P}$: effective plastic strain
*A*: yield stress of the material under reference conditions
*B*: strain hardening constant
*C*: strengthening coefficient of strain rate
*n*: strain hardening coefficient
*m*: thermal softening coefficient
${\dot{\underline{\varepsilon }}}^{P}$: effective plastic strain rate
${\dot{\bar{\varepsilon }}}_{o}$: reference strain rate
  (3) $$\hat{T}\{\begin{array}{c} 0\;\;\;\;\mathrm{for}\;T<T_{r}\\ \frac{T-T_{r}}{T_{m}-T_{r}}\;\;\;\mathrm{for}\;T_{r}\leq T\leq T_{m}\\ 1\;\;\;\mathrm{for}\;T>T_{m} \end{array}$$
  where $T_{r}=T_{room}$ corresponds to the reference temperature, $T_{m}=1793\;K$, and *T* is the deformation temperature; ${\dot{\bar{\varepsilon }}}_{o}=1\;s^{-1}$.

The Johnson–Cook parameters were calculated using the method proposed by the authors [22]. The method is shown below.

• Strain Hardening

By rearranging for $\dot{\varepsilon }=1\;s^{-1}$ and room temperature, Equation (2) can be remodeled as shown below (Equation (4)):

(4) $$\sigma^{o}=\left(A+B{\left(\varepsilon^{P}\right)}^{n}\right)$$

The values of *B* and *n* are calculated by fitting Equation (4) to the curve in Figure 2 using least-squares based optimization.

• Strain Rate Hardening

The strain rate hardening parameter, *C*, is obtained from the dynamic compression curves at room temperature ($T_{room}$) and ${\dot{\bar{\varepsilon }}}^{P}>{\dot{\bar{\varepsilon }}}_{o}$. With these conditions, the J–C equation is simplified, as shown in Equation (5).

(5) $$\sigma =\left(A+B{\left(\varepsilon^{P}\right)}^{n}\right)\cdot \left(1+C\cdot \log\left(\frac{{\dot{\bar{\varepsilon }}}^{P}}{{\dot{\bar{\varepsilon }}}_{o}}\right)\right)$$

Equation (5) can be rewritten as Equation (6).

(6) $$\frac{\sigma }{\left(A+B{\left(\varepsilon^{P}\right)}^{n}\right)}=\left(1+C\cdot \log\left(\frac{{\dot{\bar{\varepsilon }}}^{P}}{{\dot{\bar{\varepsilon }}}_{o}}\right)\right)$$

Using the values of *A*, *B*, and *n* previously calculated, $\frac{\sigma }{\left(A+B{\left(\varepsilon^{P}\right)}^{n}\right)}$ vs. $\left(\ln\frac{{\dot{\bar{\varepsilon }}}^{P}}{{\dot{\bar{\varepsilon }}}_{o}}\right)$ is plotted for 2700, 3700, and 4300 $s^{-1}$. The strain rate hardening parameter, *C*, is obtained by applying least-squares based fitting to Equation (6).

• Temperature Softening

Finally, the temperature softening parameter, *m*, is calculated using the dynamic compression curves at $T>\;T_{room}$ and ${\dot{\bar{\varepsilon }}}^{P}>\;{\dot{\bar{\varepsilon }}}_{o}$. The Johnson-Cook equation is written as is shown in Equation (7):

(7) $$\bar{\sigma }\left({\bar{\varepsilon }}^{P},\dot{{\bar{\varepsilon }}^{P}},\;T\right)=K\left[1-\theta^{m}\right]$$

where $K=\left(A+B{\left({\bar{\varepsilon }}^{P}\right)}^{n}\right)\cdot \left(1+C\cdot \log\left(\frac{{\dot{\bar{\varepsilon }}}^{P}}{{\dot{\bar{\varepsilon }}}_{o}}\right)\right)$. By applying the logarithm to both sides, Equation (7) is rewritten as in Equation (8).

(8) $$\ln\left(K-\bar{\sigma }\right)=m\;\ln\theta +\ln K$$

The temperature sensitivity is obtained by applying least-squares based fitting.

#### 2.2.4. Analysis of Thermo-Viscoplastic Behavior under Different Rates and Temperatures

The values obtained for the Johnson–Cook parameters and other properties of the tested NICRO 12.1 steel plates are shown in Table 4.

The calculated Johnson–Cook parameters were used to develop a numeric model in ABAQUS/Explicit to simulate the impact behavior of NICRO 12.1 steel plates. Different values of ${\bar{\varepsilon_{f}}}^{p}$ were used. The chosen value of ${\bar{\varepsilon_{f}}}^{p}$ for each projectile nose shape provides a better adjustment of the simulated ballistic curve to the experimental curve.

**Figure 6:**
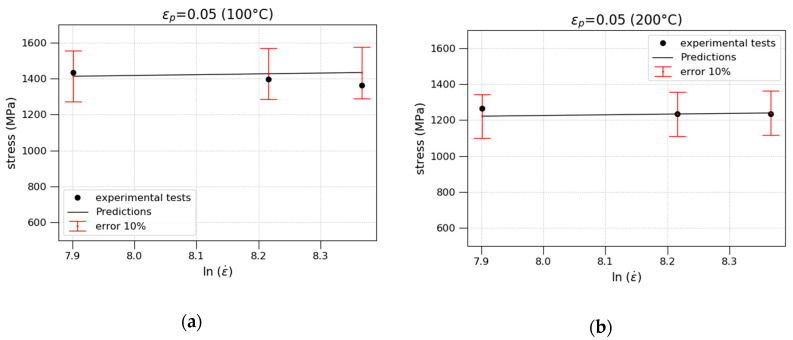
Stress as a function of strain rate: (**a**) 100 °C; (**b**) 200 °C.

In Figure 6, the stress vs. $\ln\dot{\varepsilon }$ at 100 and 200 °C for $\varepsilon_{p}=0.05$ is plotted. As depicted, the results obtained present an error lower than 10%.

#### 2.2.5. Impact Tests

**Figure 7:**
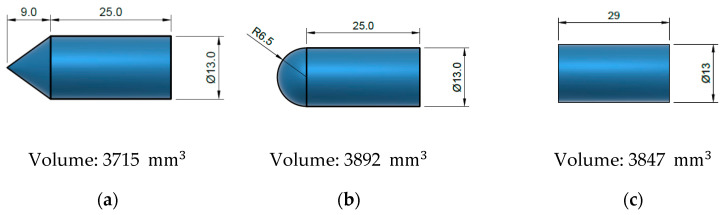

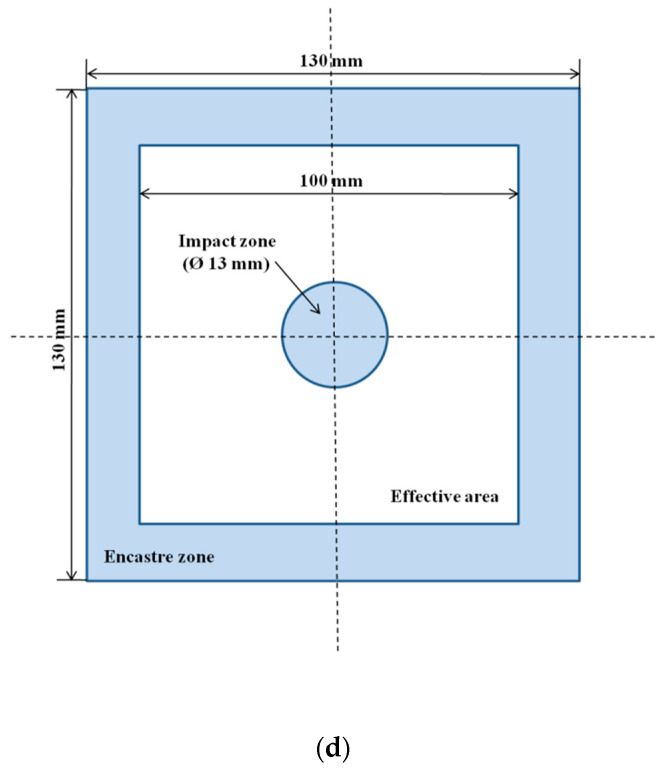
Geometry and dimensions in mm of (**a**) conical, (**b**) hemispherical, and (**c**) blunt projectiles. (**d**) Plate sketch. Dimensions in mm.

NICRO 12.1 steel plates of 130 × 130 ${\mathrm{mm}}^{2}$ and thickness of 1 mm were tested under ballistic impacts. To conduct the impact testing, a launcher device of compressed air with a gun of tubular geometry with diameter of *d* = 13 mm were used. The plate was placed on a rigid support with an effective area of $A_{f}$ = 100 × 100 ${\mathrm{mm}}^{2}$, as shown in Figure 7d. In addition, the projectiles were launched at the center of the plate. This area is called the impact zone in Figure 7d.

**Figure 8:**
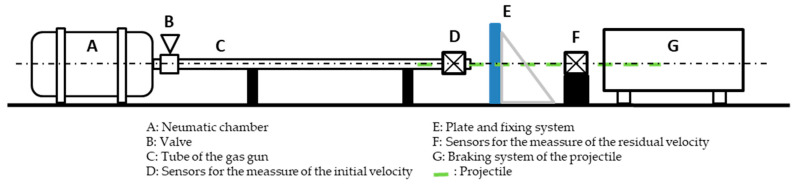
Experimental set-up for the ballistic characterization.

Following the set-up shown in Figure 8, a temporal signal was registered once the projectile crossed through the lasers system coupled to photodiodes and temporal counters. This procedure was repeated two times. The two times prior to impact were used to determine the initial impact velocity ($v_{0}=\Delta X_{12}^{laser}/\Delta t_{12}$) and the two times after impact were used to determine the residual impact velocity ($v_{r}=\Delta X_{34}^{laser}/\Delta t_{34}$). $\Delta X_{ij}$ is the known distance between the lasers *i* and *j*, and $\Delta t_{ij}$ is the time range registered between the lasers *i* and *j*.

The ballistic performance of the steel was analyzed using three types of projectiles: conical, blunt, and hemispherical. The dimensions of the projectiles are shown in Figure 7a–c. The production tolerance was ±0.1 for each of the projectiles (ballistic results were corrected with the Lambert–Jonas equation [25]; thus, production tolerances were avoided). The mass of the projectiles was 30 g to keep the same kinetic energy. Their trajectories were kept perpendicular to the plate in all cases. The impact velocities tested were in the range 75 to 200 m/s.

**Figure 9:**
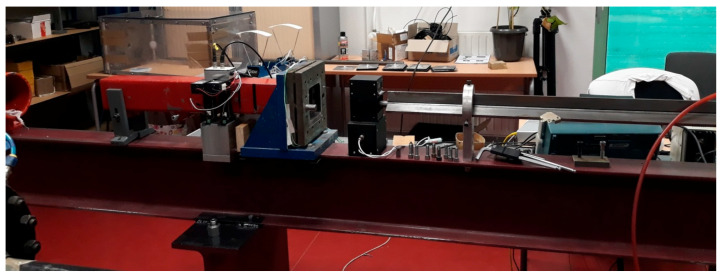
Experimental device used for impact tests.

The experimental device used for the impact tests is shown in Figure 9, where the tube of the gas gun is to the right of the picture, and the sample support and braking system are to the left.

#### 2.2.6. Numerical Model

**Figure 10:**
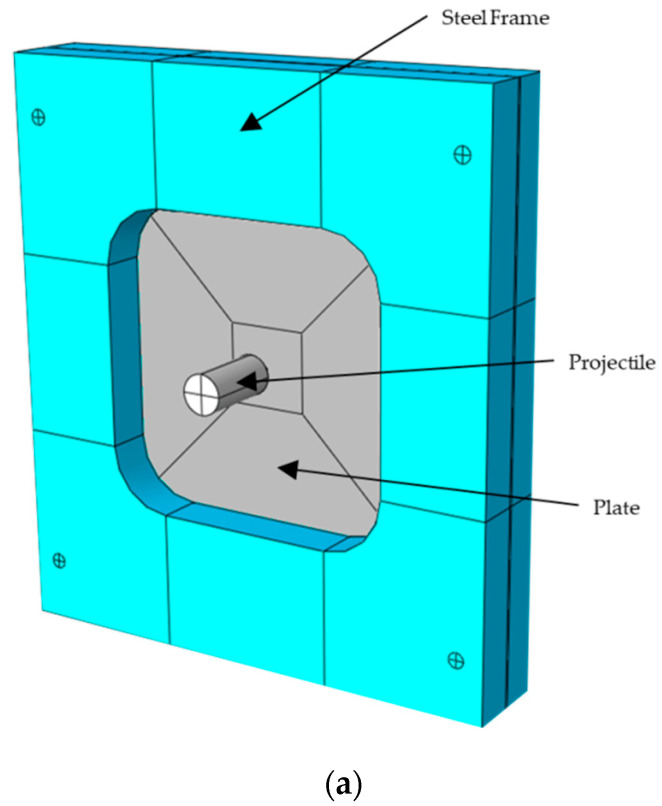

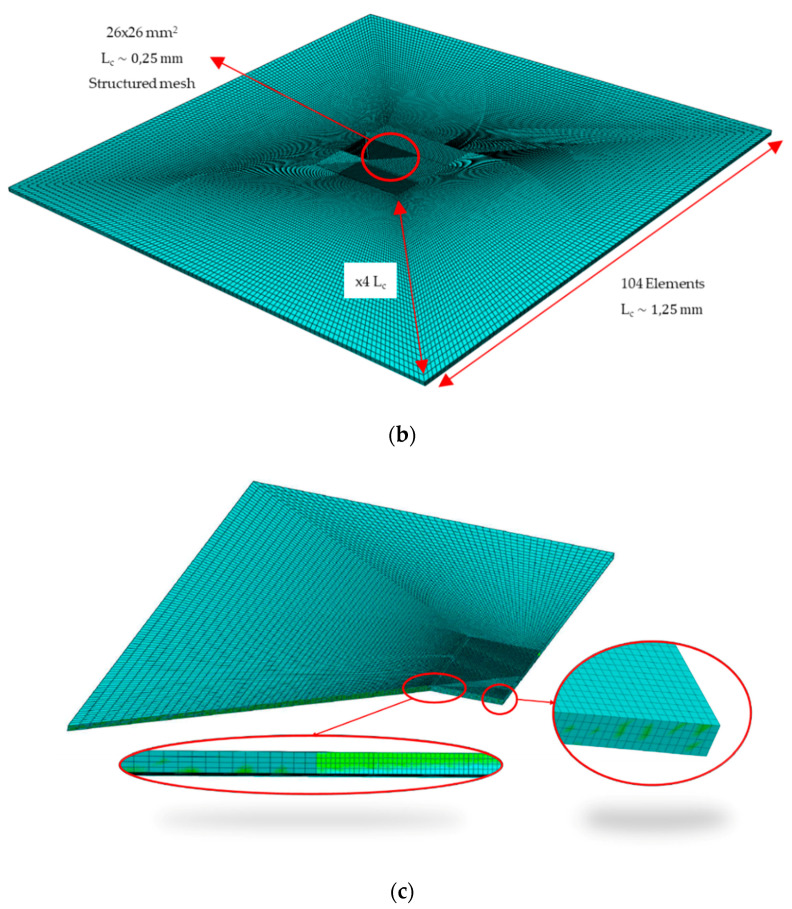
Numerical model: (**a**) assembly; (**b**) mesh; (**c**) inner part of the specimen and details.

A three-dimensional numerical model was developed to simulate the behavior of NICRO 12.1 steel plates under impact tests. The plate was modeled as a homogeneous solid of 130 × 130 ${\mathrm{mm}}^{2}$ and thickness of 1 mm. The J–C model was used to model the thermo-viscoplastic behavior of NICRO 12.1 steel, using the parameters obtained in the previous sections (Table 4). The assembly is shown in Figure 10a.

The projectile was modeled as a rigid body because no plastic deformation or erosion were observed during the impact tests. The mass of each projectile was 30 g, and an equivalent density was used for each projectile to obtain their exact mass. The equivalent densities of blunt, conical, and hemispherical projectiles were 7745, 7761, and 7420 $\mathrm{kg}/m^{3}$, respectively.

The plate was attached to a steel frame that supported the sample. This steel frame was 190 × 180 × 30 ${\mathrm{mm}}^{3}$ and had a 100 × 100 ${\mathrm{mm}}^{2}$ inner cut-out, which corners were rounded with a 20 mm fillet to reduce stress concentration in this zone. The frame was modeled using a linear elastic solid, in which density, elastic modulus, and Poisson coefficient were 7850 $\mathrm{kg}/{\mathrm{mm}}^{3}$, 210 GPa, and 0.33, respectively.

The plate presents two different meshed zones (Figure 10b,c) so that an equilibrium between the computational time, minimizing the errors due to the mesh size could be found [26]. A central squared zone of 26 × 26 ${\mathrm{mm}}^{2}$ (double the projectile diameter) contained mesh structured with an element size of 0.25 mm. Within this zone, the element size increased gradually up to ×4 size. The free edges of the plate were meshed with 100 elements. The thickness direction was meshed with four elements. The plate was meshed with 3D solid hexahedral elements with reduced integration and Enhanced Hourglass Control with 143,104 elements and 179,925 nodes.

**Figure 11:**
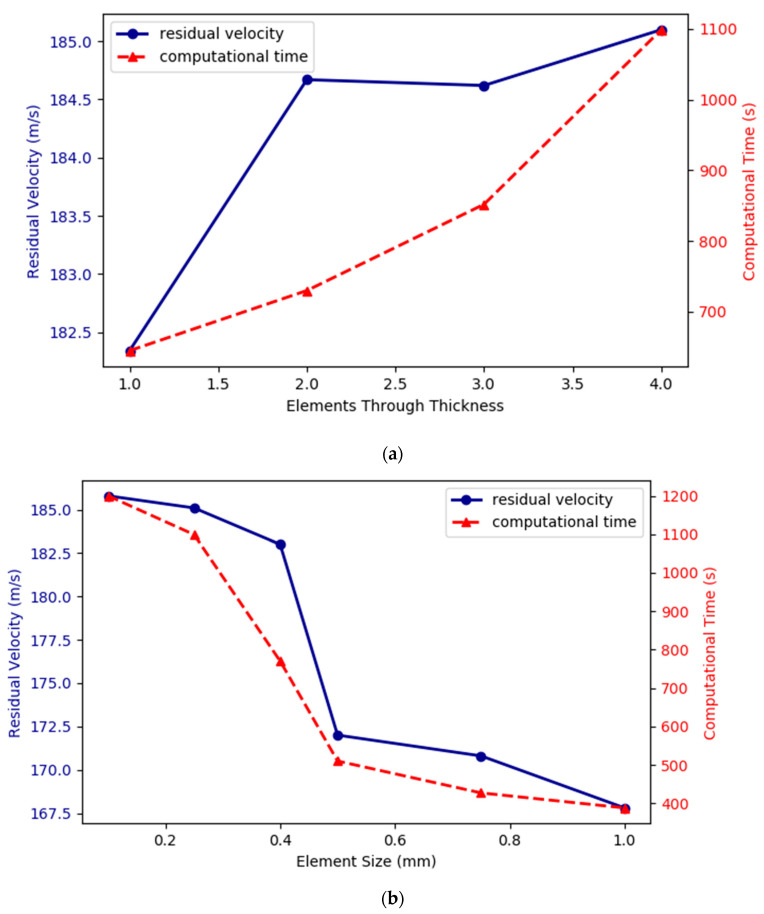
Mesh sensitivity for: (**a**) the number of elements in the thickness direction; (**b**) element size.

A mesh sensitivity analysis of element size in the central zone and the number of elements in the thickness direction was carried out to select the optimum element size, which combines good accuracy on results with acceptable computational cost. This analysis was conducted using blunt projectile and $v_{0}=200\;m/s$. The element size was held constant (0.25 mm), and the number of elements in the thickness direction was changed from 1 to 4 (see Figure 11a). In addition, the element size was tested (see Figure 11b) in a range from 0.1 to 1 mm, with a constant number of elements in the thickness direction (four elements). Due to the results of the sensitivity study shown in Figure 11, an element size of 0.25 mm and 4 elements in the thickness direction were chosen.

**Figure 12:**
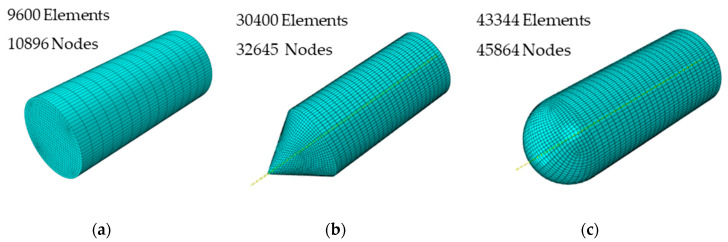
Mesh of the projectiles: (**a**) blunt, (**b**) conical, and (**c**) hemispherical.

The steel frame was meshed using hexahedral elements with reduced integration with 68,850 elements and 77,972 nodes. Each part was meshed using hexahedral elements with reduced integration. The mesh detail is shown in Figure 12.

A general contact interaction based on “hard contact” interaction was imposed to avoid element interpenetration, and for tangential behavior, a penalty friction coefficient (μ) equal to 0.15 [27] was selected. The frame was clamped in all degrees of freedom around the exterior perimeter to simulate the attachment conditions of the experiments during the impact event.

#### 2.2.7. 3D Scanner

**Figure 13:**
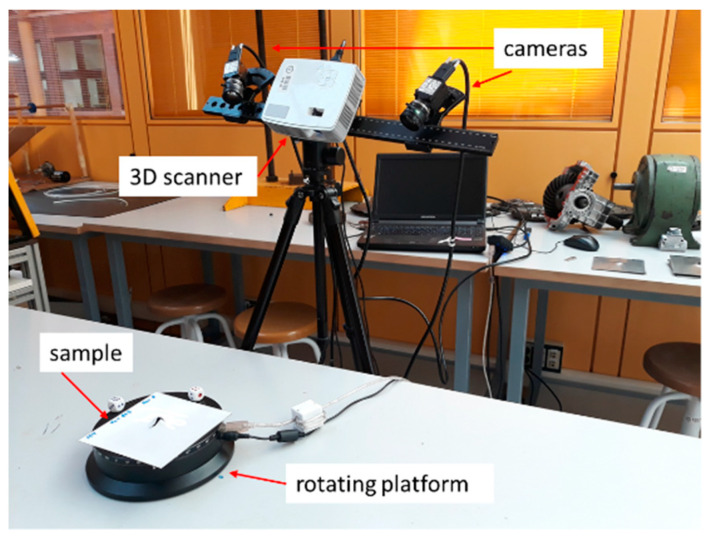
HP 3D scanner test.

After the ballistic impacts, an HP (Hewlett-Packard) brand scanner (Figure 13) was used to measure the surface damage and the local and global deformation of the tested plates. The equipment was composed of two HP high definition cameras for stereoscopic image capturing combined with a structured light projector. This creates a black and white pattern (structured light) and projects it on the object. An automatic 360 rotating platform allows the sample to be placed and rotated, while the cameras capture the light reflected on it [28].

Table 5, provides the scanner characteristics.

The scanned images must be aligned to obtain the final shape representation. Then, this was exported to Geomagic Design X for post-processing of the image (i.e., visualization of the dimensions and defects on the surface) [28].

## 3. Numerical and Experimental Results

### 3.1. Ballistic Impact Results

**Figure 14:**
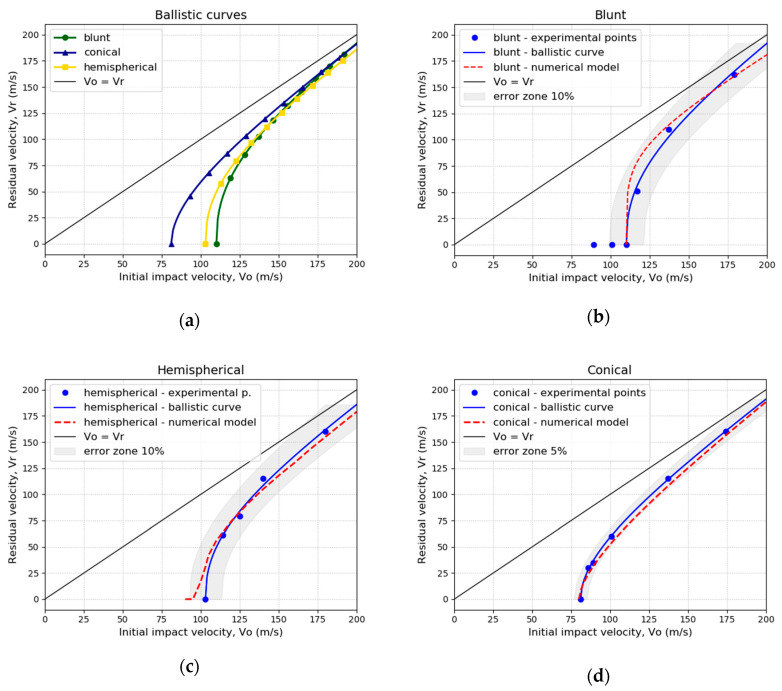
Ballistic curves: (**a**) experimental curves; (**b**) blunt projectile; (**c**) hemispherical projectile; (**d**) conical projectile.

Figure 14 shows the residual impact velocity versus initial impact velocity ($v_{r}-v_{0}$) curves obtained with each projectile: blunt, conical, and hemispherical. In addition, an error zone is plotted for each projectile. This zone corresponds to a percentage of error calculated with respect to the variation in $v_{0}$ for each one.

For the conical projectile, the ballistic limit obtained is $v_{0}=80\;m/s$. This is clearly smaller than the ballistic limit obtained with the hemispherical projectile, $v_{0}=105\;m/s$, and the blunt projectile, $v_{0}=115\;m/s$. In contrast, for $v_{0}$ higher than 175 m/s, the curves of the three projectiles converge to the same $v_{r}$ (Figure 14a). Beyond 175 m/s, mass inertial effects are important, i.e., no dependence on the projectile geometry is found in the residual impact velocity. This behavior has been observed in other research [5,29].

The experimental results were compared with the numerical simulations. The maximum difference in $v_{r}$ (residual impact velocity) between the experimental and the numerical results is about 10% for a blunt (Figure 14b) and hemispherical (Figure 14c) projectiles and 5% for the conical projectile (Figure 14d).

### 3.2. Failure Modes

The local and global deformations of the material yield relevant information. The local deformations correspond to the mode of failure that the projectile produces when impacting the plate. In this case, the HP 3D scanner was used to measure the induced deflection in the plate according to the projectile nose shape and the initial impact velocity.

**Figure 15:**
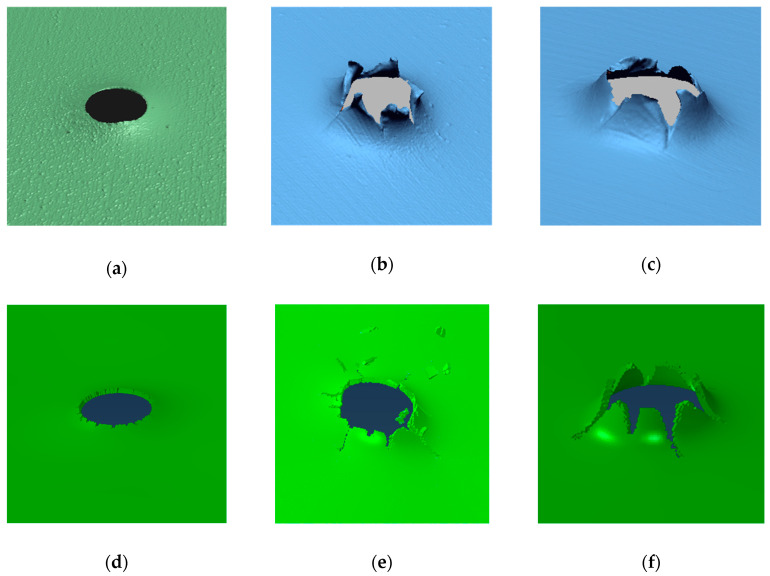
Failure experimental mode: (**a**) blunt, (**b**) conical and (**c**) hemispherical. Failure numerical mode: (**d**) blunt, (**e**) conical and (**f**) hemispherical.

The failure mode of the sheet steel was analyzed. It was found that the projectile nose shape has a strong influence on the process of failure. For the blunt projectile (Figure 15a), plug ejection was observed due to high shearing during the failure process. In contrast, during the perforation with a conical projectile (Figure 15b), the material target was moved to the side causing radial flow, necking due to piercing, and finally, the creation of petals. The number of petals was kept constant, around five, for all of the tested velocities.

The hemispherical penetrator (Figure 15c) moved the material target forward leading to the formation of shear bands that induced circumferential necking, followed by a plug ejection. Finally, petals appeared on the back of the plate. This process requires more plastic work than in the blunt case.

The simulations of plates impacted with blunt projectiles present plugging failure, as shown in Figure 15d. In contrast, the simulated plates impacted with conical and hemispherical projectiles present petaling failure (Figure 15e,f). However, in the case of the hemispherical projectile, the petals obtained are more prominent than in the case of the conical projectile, and a small plugging occurs due to a circumferential necking, whereas it does not appear with the conical projectile.

A correspondence exists between the simulated and experimental failure mode for each projectile nose shape. Furthermore, the same behavior was observed in other studies, as mentioned in the introduction.

### 3.3. Maximum Plate Deformations

**Figure 16:**
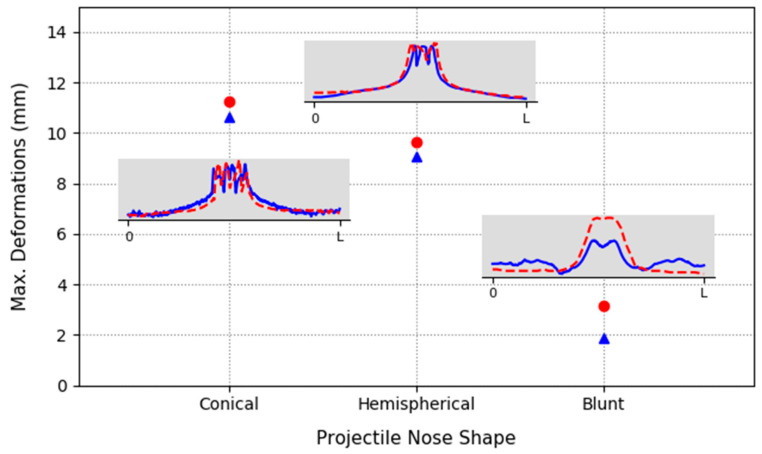
Maximum deformations for each projectile: circles—discontinued line (experimental measurements), triangles—continued line (numerical model).

The examination of the local and global deformations of the material is relevant in the behavior of materials under the impact. The global deformations consist of the evaluation of the deflection profile of the plate after impact. The tested plates were scanned with the HP 3D scanner, and Geomagic Design X software was used to measure the experimental deformation for each element of the plate, defined as the distance between the position before and after the impact. In addition, the numerical measures were obtained from the simulations. The experimental and numerical results for the maximum deflections and the profiles are plotted in Figure 16.

The maximum deflection observed with the blunt projectile is 3.18 mm in the experimental test and 1.89 mm in the numerical test. The plastic deformation is highly localized around the impacted zone, due to the small thickness of the sheet, and small plastic deformation is observed in the rest of the plate.

The maximum deformation measured experimentally with the conical projectile is 11.27 mm, while the value obtained from the numerical profile is 10.64 mm.

In the impact with the hemispherical projectile, the maximum deformation obtained is 9.64 mm and 9.08 mm for experimental and numerical tests, respectively.

## 4. Discussion

The ballistic curve for each projectile was plotted observing different ballistic limits for each projectile nose shape. The blunt projectile presented the highest ballistic limit. The ballistic limit with the hemispherical projectile was slightly lower than that of the blunt projectile. The ballistic limit for the conical projectile was the lowest. The dependence on the projectile nose shape observed in the ballistic limit is due to the stress state induced by each projectile.

It was observed that the failure mode is strongly correlated to the nose projectile. With the blunt projectile, the failure mode observed was plugging, while petaling was the failure mode observed with the conical projectile, and petaling and small plug ejection appeared with the hemispherical projectile.

The maximum deformation with each projectile was studied and observed to be strongly linked to the nose projectile. The maximum deformation was obtained with the conical projectile, was slightly lower with the hemispherical projectile, and decreased significantly with the blunt projectile.

Numerical simulations were performed and calibrated with ABAQUS/Explicit. The numerical model allowed prediction of the ballistic behavior of the NICRO 12.1 steel sheets with a thickness of 1 mm. The developed model gave excellent predictions of ballistic limit, failure mode, and permanent deformations of the plate after the impacts. There is a close correlation between these with the three projectiles tested (blunt, conical, and hemispherical) with less than 10% deviation in all of the cases.

The results obtained in this work can be used in the numerical modeling of engineering problems involving impact loadings in NICRO 12.1

## 5. Conclusions

In this work, NICRO 12.1 steel plates were characterized in terms of ballistic impact with three nose projectiles (blunt, conical, and hemispherical) and speed up to 200 m/s. Quasi-static tensile and dynamic compression tests were performed at a large range of temperatures and strain rates. The results were used to calculate the J–C parameters. A numerical model was developed to predict the impact ballistic behavior of NICRO 12.1 using the J–C model. Ballistic curves, failure mode, and the maximum deformation were obtained experimentally and numerically.

For each projectile, the ballistic curves were obtained with a good correlation between experimental and numerical results. Different penetration powers were observed for each, and the following ballistic limits were obtained: $v_{0}=80\;m/s$ for the conical projectile, $v_{0}=105\;m/s$ for the blunt projectile, and $v_{0}=115\;m/s$ for the hemispherical projectile.

In the case of the failure mode, a small amount of plug ejection was produced, and petals appeared with the hemispherical projectile, petaling was observed with the conical projectile, and plugging was observed with the blunt projectile.

In addition, the maximum deformations were measured for each projectile. The highest deformations were obtained for the conical projectile, then for the hemispherical projectile, and finally for the blunt projectile.

## Figures and Tables

**Table 1:** Chemical composition of NICRO 12.1. Data from [18].

Fe	C	Cr	Ni	Si	Mn
74.15%	Max. 0.15%	17%	7%	0.7%	1%

**Table 2:** Chemical composition of annealed and pickled AISI 301 (mas %). Data from [19].

**C**	**Mn**	**P**	**S**	**Si**	**Cr**	**Ni**	**N_2_**	**Co**
0.02	1.57	0.023	0.003	0.41	17.41	6.6	0.119	0.12
**Cu**	**Mo**	**Sn**	**Ti**	**Al**	**Nb**	**O**	**B**	**Fe**
0.09	0.05	0.009	0.009	0.005	0.009	0.0074	0.0017	Balance

**Table 3:** NICRO 12.1 properties.

$\rho \;[\mathrm{kg}/{\mathrm{cm}}^{3}]$ ^1^	*E* [GPa] ^2^	*ν* ^3^	$T_{m}\;[K]$ ^4^	$c_{p}\;[J/\mathrm{kgK}]$ ^5^
0.0079	205	0.29	1793	477

^1^ density, ^2^ Young’s modulus, ^3^ Poisson coefficient, ^4^ melting temperature, ^5^ heat capacity at constant pressure.

**Table 4:** Johnson–Cook parameters.

***A* [MPa]**	***B* [MPa]**	***C***	***n***	***m***
1055	469	0.013	0.27	1.13
***E* [GPa]**	***ν***	$c_{p}$ **[J/kgK]**	$T_{m}$ **[K]**	$T_{r}$ **[K]**
205	0.29	477	1793	300
${\dot{\bar{\varepsilon }}}_{o}\;\left[s^{-1}\right]$	${\bar{\varepsilon_{f}}}^{p}$ **blunt**	${\bar{\varepsilon_{f}}}^{p}$ **conical**	${\bar{\varepsilon_{f}}}^{p}$ **hemispherical**	
1	0.2	0.16	0.29	

**Table 5:** HP brand scanner characteristics.

Scanner Model:	HP 3D Structured Light Scanner Pro S3
Resolution:	Up to 0.08 mm
Minimum scan time for single image capture:	2 s
Light projector:	ACER, model K132
